# The roles, training and knowledge of community health workers about diabetes and hypertension in Khayelitsha, Cape Town

**DOI:** 10.4102/curationis.v41i1.1815

**Published:** 2018-03-26

**Authors:** Lungiswa P. Tsolekile, Helen Schneider, Thandi Puoane

**Affiliations:** 1 School of Public Health, University of the Western Cape, South Africa; 2 SAMRC Health Services to Systems Unit, University of the Western Cape, South Africa

## Abstract

**Background:**

The current roles and capacity of community health workers (CHWs) in the management and control of non-communicable diseases (NCDs) remain poorly understood.

**Objectives:**

To assess CHWs’ current roles, training and knowledge about diabetes and hypertension in Khayelitsha, Cape Town.

**Methods:**

A cross-sectional study of 150 CHWs from two non-governmental organisations contracted to provide NCD care as part of a comprehensive package of services was conducted. An interviewer-administered closed-ended questionnaire was used to determine the roles, training, in-service support, knowledge and presence of NCDs. Descriptive analyses of these domains and multivariate analyses of the factors associated with CHWs’ knowledge of hypertension and diabetes were conducted.

**Results:**

The vast majority (96%) of CHWs were female, with a mean age of 35 years; 88% had some secondary schooling and 53% had been employed as CHWs for 4 years or more. Nearly half (47%) reported having an NCD. CHWs’ roles in NCDs included the delivery of medication, providing advice and physical assessment. Only 52% of CHWs reported some formal NCD-related training, while less than half of the trained CHWs (*n* = 35; 44%) had received follow-up refresher training. CHWs’ knowledge of diabetes and hypertension was poor. In the multivariate analyses, higher knowledge scores were associated with having an NCD and frequency of supervisory contact (≥1 per month).

**Conclusions:**

The roles performed by CHWs are broad, varied and essential for diabetes and hypertension management. However, basic knowledge about diabetes and hypertension remains poor while training is unstandardised and haphazard. These need to be improved if community-based NCD management is to be successful. The potential of peer education as a complementary mechanism to formal training needs as well as support and supervision in the workplace requires further exploration.

## Introduction

Non-communicable diseases (NCDs) such as diabetes and hypertension are public health problems that place a heavy burden on health facilities. To address this burden and to provide continuity of care for NCDs, it is increasingly recommended that services be offered outside of health facilities in communities (Ndou et al. 2013). In South Africa, community health workers (CHWs) provide community-based care, but for years, their efforts have been focused on home-based care for people with HIV and TB, with little done in the area of NCDs. There is a scarcity of information describing the processes required to integrate NCD care into the work of generalist CHWs, including training, supervision and orientation into roles. Where it has been assessed, studies have suggested that CHWs lack essential knowledge of chronic diseases (Bradley & Puoane 2007; Sengwana & Puoane 2004).

This study sought to fill this gap and shed light on the roles of CHWs in diabetes and hypertension management in an urban area of the Western Cape. Based on current practices, it suggested possible avenues for capacity building of CHWs for NCD care in South Africa, especially in resource-constrained settings.

This study assessed the roles, training, in-service support and knowledge related to NCDs (i.e. diabetes and hypertension). In addition, the study also examined factors associated with knowledge among CHWs providing NCD care in an urban township. It was done with the purpose of informing the development of appropriate and context-sensitive CHW training programmes. The questionnaire in this study assessed the following:

- the socio-demographic profile and presence of NCDs among CHWs
- roles of CHWs in NCD management and prevention
- types of training received by CHWs on NCDs and frequency of supervision
- community health workers’ knowledge of risk factors, complications and prevention of diabetes and hypertension.

### Definition of key concepts

Community health workers are lay workers who have no professional training but have some training in the context of the intervention and are responsible for delivering services related to health care (Lewin et al. 2005).

### Background and literature review

Non-communicable diseases such as diabetes and hypertension have contributed significantly to the burden of disease globally, including in South Africa (Mayosi et al. 2009). According to the International Diabetes Federation (IDF), 2.28 million South Africans had diabetes in 2015 (IDF 2015), while the prevalence of hypertension is on the increase (Gomez-Olive et al. 2013; Lloyd-Sherlock et al. 2014; Thorogood et al. 2007). According to the World Health Organization in 2015, three quarters of all NCD-related deaths now occur in low- and middle-income countries (LMIC) (WHO 2016), where diabetes prevalence quadrupled between 1980 and 2014 (NCD Risk Factor Collaboration 2016). This rise in NCDs has implications for health systems and populations, affecting the most economically active age groups (Islam & Biswas 2014; Peck et al. 2014). In many regions of the world, health systems are ill-prepared to deal with the problem, and the prevention and management of NCDs remain a significant challenge. One of the constraints to managing NCDs is the poor availability of adequately trained human resources (Islam & Biswas 2014; Peck et al. 2014).

Community health workers have been proposed as a strategy to mitigate the shortage of health care professionals (Lehmann & Sanders 2007; Liu et al. 2011; O’Brien et al. 2009) such as nurses and doctors. CHWs’ relationship with the community, as well as their understanding of the context, culture and language, puts them in an ideal position to assist communities with health-related issues (Lehmann & Sanders 2007; Lehmann et al. 2009). Furthermore, CHW programmes provide a link with the formal health care system, thereby ensuring a continuum of care (Liu et al. 2011), vital for longevity and the prevention of complications of NCDs.

Studies have shown that CHWs can play a valuable role in the prevention, management and care of chronic lifelong (CLL) conditions such as human immunodeficiency virus (HIV) and acquired immunodeficiency syndrome (AIDS) (Callaghan, Ford & Schneider 2010; Mwai et al. 2013; Schneider, Hlophe & Van Rensburg 2008). The role of the CHWs has also been cited in the prevention and management of diabetes and hypertension in LMIC (Farzadfar et al. 2012; Jafar et al. 2010). In these contexts, CHWs have shown to be effective in providing education as well as support to people with NCDs (Bradley & Puoane 2007; Gaziano et al. 2015; Ndou et al. 2013). There is also considerable evidence on the role and effectiveness of CHWs for the prevention and management of NCDs in high-income countries, where roles similarly include health education, adherence support and counselling (Brownstein et al. 2005, 2007; Cherrington et al. 2008). However, for CHWs to perform these tasks, they require appropriate training (Abrahams-Gessel, Denman & Montano 2015b; Lopes, Cabral & De Sousa 2014), supportive supervision (Källander et al. 2015), materials and equipment. Despite the potential role of CHWs in NCDs, studies indicate that CHWs often have poor knowledge about NCDs and their risk factors (Abrahams-Gessel, Denman & Montano 2015a; Puoane, Bradley & Hughes 2006).

Community health workers in many parts of South Africa are employed by non-governmental organisations (NGOs) intermediaries, which are often contracted by the government to render services to communities (Van Pletzen et al. 2014). These NGOs are responsible for the capacity development of the CHWs, including their training needs, orientation into roles and supervision. Overall, community-based approaches to NCDs are underdeveloped, inadequately defined and fragmented. However, in South Africa, a diverse community-based care and support infrastructure based on lay health workers is being reorganised into a more comprehensive system of outreach. Under a set of initiatives referred to as PHC Re-engineering (Naledi, Barron & Schneider 2011), a formalised CHW cadre has been piloted with expanded roles. These roles extend beyond HIV and tuberculosis (TB) to include maternal child health interventions and integrated approaches to chronic lifelong conditions (involving both communicable and NCDs).

This cadre of workers will be organised into ward-based outreach teams (WBOTs) supervised by a nurse and supported by health facilities. In the first phase of primary health care (PHC) re-engineering, the focus has been on developing the capacity for maternal child health (Whyte 2015).

## Methods

A cross-sectional study of CHWs employed by two NGOs providing NCD care in Khayelitsha, Cape Town, was conducted.

### Population and sampling

Khayelitsha is a large informal settlement in Cape Town. In 2011, this township had an estimated population of 391 749 people (Statistics South Africa 2012). At the time of the study (2013), community-based services were provided by a network of 5000 CHWs employed by 45 NGOs, which were predominantly funded by government departments such as health and social development (Van Pletzen et al. 2014). However, only three NGOs were supported and funded by the Ministry of Health to provide NCD care in the community. All three NGOs were purposively selected and approached for the study, two of which consented to participate. All 160 CHWs employed in the two NGOs were then approached for an interview.

### Data collection

Trained research assistants using mobile phones administered a questionnaire in isiXhosa (local language). Data were collected in 2013 for 2 months.

The development of the questionnaire involved an initial phase where CHWs were observed conducting their daily activities. The observations of practice provided insights about CHWs scope of practice and work organisation among other things. This formative, exploratory phase, which is reported elsewhere (Tsolekile et al. 2014), provided the basis for designing the questionnaire for this study. Findings from this formative phase resulted in the development of four critical areas (constructs) within the CHWs’ sphere of activity related to diabetes and hypertension: roles, training and induction, supervision and support, and knowledge. In an iterative process, the co-authors developed a questionnaire to assess these activities quantitatively.

Regarding their roles, CHWs were asked the following question (without further probing): ‘What are the services that you provide to clients with hypertension and diabetes?’ The responses were entered into a pre-coded list by fieldworkers. Knowledge was similarly assessed through a series of closed-ended questions covering risk factors, complications and prevention of hypertension and diabetes. There were 26 and 27 possible knowledge responses about diabetes and hypertension, respectively. These were basic and appropriate to the scope of CHWs.

The data collection tool was piloted to ensure the appropriateness and understanding of questions and to test its content validity. The questionnaire was piloted in another township 15 km away from the study site with 17 CHWs who also provided NCD care to community members.

### Analysis

Data analysis was performed using SPSS software, version 24 for Windows (Microsoft, USA). Univariate analysis was conducted on socioeconomic characteristics, NCD-related training, knowledge about NCD (including risk factors, complications, preventive measures) and roles of participants. Multivariate analysis was conducted to establish the relationship between the independent variables (various socioeconomic factors, training) and the dependent variables (knowledge scores for diabetes and hypertension). In all analyses, statistical significance was set at *p* < 0.05.

For each domain of knowledge, responses were scored. A score of zero was given to wrong or missing responses, and a score of one for correct answers. The means, medians and 95% confidence intervals for knowledge scores were calculated for each disease.

### Ethical consideration

Permission (No. 11/4/4) to conduct the study was obtained from the Research and Ethics Committee at the University of the Western Cape, the Provincial Department of Health and the two NGOs that took part in the study.

## Results

Of the 160 CHWs approached for an interview, 150 consented to participate, giving a response rate of 94%.

### Socio-demographic profile of CHWs

Table 1 shows that CHWs were mostly female (*n* = 144) rather than male (*n* = 6). The mean age of CHWs was 35.4 years; 88% had some secondary schooling and 36% had completed grade 12. More than half (53%, *n* = 79) of the CHWs had been in employment for 4 years and more. Seventy (47%) CHWs reported to have at least one NCD, with hypertension (33%) being the most common. Nearly one quarter of the 150 CHWs (*n* = 34, 23%) had more than one NCD referring to a combination of diseases or conditions such as diabetes, hypertension, arthritis, heart diseases, asthma and strokes.

**TABLE 1:** Demographic and self-reported non-communicable diseases characteristics of the community health workers (*n* = 150).

Variables	Frequency	Percentage
**Gender**		
Male	6	4
Female	144	96
**Age**		
less than 30 years	50	33
30–39 years	56	37
40–49 years	27	18
50 years and above	17	11
Mean age in years	35.4	-
**Educational attainment**		
No schooling	0	-
Primary schooling (Grade 1–7)	10	7
High school (Grade 8–11)	78	52
Matric (Grade 12)	54	36
Post matric qualification	8	5
Mean number of schooling (years)	10.8	-
**Duration of employment**		
Less than 1 year	32	21
1–3 years	39	26
4 or more years	79	53
**Self-reported NCD**		
Hypertension	50	33
Diabetes	36	24
Asthma	15	10
Arthritis	14	9
Stroke	11	7
Other heart conditions	5	3
**Comorbidities**		
No NCD	80	53
One NCD	36	24
More than one NCD	34	23

NCD, non-communicable diseases.

### Roles of community health workers related to non-communicable diseases (diabetes and hypertension)

**FIGURE 1:**
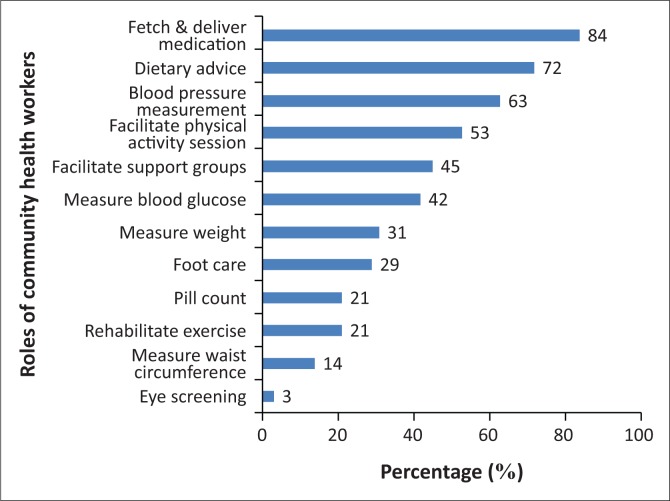
Non-communicable disease-related roles or tasks performed in the community (*n* = 150).

Community health workers offered a diversity of responses on their roles in the management and care of NCDs (Figure 1). The four most reported roles performed included the distribution of medication (84%), advising about diet (72%), measuring of blood pressure (63%) and conducting physical activity sessions (53%). Only 21% conducted pill counts as part of their routine practice.

### Training related to non-communicable diseases

Of the 150 CHWs interviewed, only 79 (52%) reported having received formal NCD-related training, and of those, less than half (*n* = 35; 44%) received refresher training after the initial training.

**FIGURE 2:**
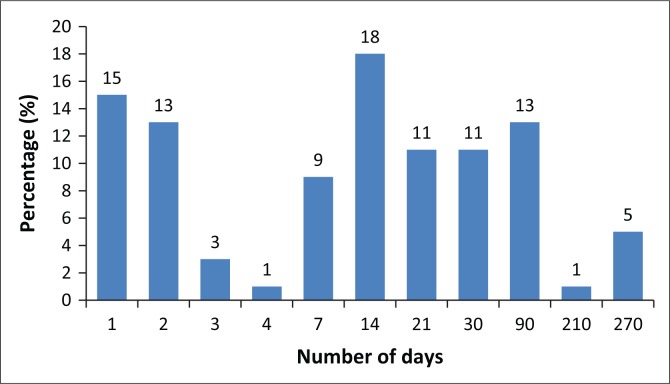
Duration of non-communicable disease-related training among community health worker (*n* = 79).

A wide variety of training experiences and providers were reported, the duration of which ranged from 1 to 270 days (9 months) (Figure 2). More than half of the 79 respondents (*n* = 46; 58%) reported training of 14 days or less, mostly 1 or 2 days (28%), while the remainder indicated more extended periods. Training providers were a mix of higher education institutions (often as part of research), the Department of Health and independent trainers.

Although 48% (*n* = 71) of the CHWs had no NCD-related training, they reported being orientated into their NCD-related roles by supervisors (nurses) (31%), NGO coordinators (34%) and fellow CHWs (25%).

### In-service supervision and support

The frequency of supervision of CHWs varied from engaging the supervisor once a month (25%) to more than three times a month (*n* = 93; 62%) (Table 2). Observed supervision (supervisor accompanying CHWs on the job) was commonly reported, with only 10% of CHWs not remembering when last they were observed performing their tasks. A vast majority (91%) of CHWs viewed their supervisors as being very supportive or supportive.

**TABLE 2:** In-service supervision and support (*n* = 150).

Variables	Frequency	Percentage
**Frequency of meetings with supervisor monthly**		
Once	38	25
Twice	4	3
Three times	13	9
More than thrice	93	62
It depends	2	1
**Frequency of observing tasks performed**		
This week	51	34
Last week	22	15
Sometime this month	10	7
Last month	37	25
Two months ago or more	15	10
Do not remember	15	10
**Level of supervisor support**		
Very supportive	122	81
Supportive	15	10
Ambivalent about support	2	1
Unsupportive	7	5
Very unsupportive	4	3

### Knowledge related to diabetes and hypertension

Knowledge of diabetes and hypertension among CHWs was poor, with mean scores being one third of the expected knowledge scores (Table 3). Scores for preventive measures were slightly better than for the risk factors and complications.

**TABLE 3:** Community health workers knowledge scores of diabetes and hypertension.

Type of NCD	Total value	Min. score	Max. score	Mean score	Median	95% CI	
						Lower	Upper
**Diabetes**							
Risk factors	9	0	5	1.81	2	1.83	2.36
Complications	6	0	6	2.09	2	1.62	2.01
Preventive measures	11	1	10	4.33	4	3.97	4.70
Total score	26	2	21	8.24	7	7.50	8.98
**Hypertension**							
Risk factors	10	0	8	3.07	3	2.73	3.40
Complications	6	0	4	1.63	1	1.42	1.85
Preventive measures	11	1	10	4.8	5	4.40	5.20
Total score	27	2	22	9.50	8	8.64	10.36

NCD, non-communicable diseases; CI, confidence interval.

Multiple regression analyses were performed to ascertain the combined effects of any NCD training, age, years of schooling, NCD status (absence vs. presence of NCDs), duration of employment (≤ 3 years vs. > 3 years), and frequency of supervision (< 1 per month vs. ≥ 1 per month) on diabetes and hypertension knowledge scores (as a continuous variable).

Having an NCD and regular supervision (≥ 1 month) were strongly associated with both diabetes and hypertension knowledge scores (Table 4). The odds of knowing about diabetes were 5.4 and 4.9 times for those with an NCD or supervised more than once a month, respectively. Similar findings were observed for hypertension knowledge. Training in NCDs or years of basic schooling was not associated with improved scores.

**TABLE 4:** Multiple regression model of factors associated with diabetes and hypertension knowledge.

Variables	Knowledge of diabetes				Knowledge of hypertension			
	AOR	95% CI		*p*-value	AOR	95% CI		*p*-value
		Lower	Upper			Lower	Upper	
Age	0.667	−0.070	0.142	0.506	0.923	−0.068	0.186	0.357
Schooling years	1.625	−0.090	0.921	0.106	1.608	−0.113	1.099	0.110
NCD status (presence of NCD)	5.360	2.391	5.183	0.001	4.189	1.874	5.223	0.001
Employment (> 3 years)	−0.964	−2.221	0.765	0.337	−0.867	−2.575	1.005	0.387
Supervision (≥ 1 per month)	4.939	1.945	4.541	0.001	4.530	2.010	5.123	0.001
Training (any NCD training)	−0.134	1.945	4.541	0.894	0.627	−1.117	2.154	0.532

AOR, adjusted odds ratio; CI, confidence interval; NCD, non-communicable disease; CI, confidence interval.

## Discussion

The primary objective of this study was to assess NCD-related roles, training, and diabetes and hypertension knowledge of CHWs. In this study, CHWs had high levels of schooling, and the majority were women. In many CHW programmes, females are the preferred gender because of the type of tasks required (Jaskiewicz & Deussom 2013). Many were themselves diagnosed with an NCD. More than half of CHWs had been employed for 4 or more years, indicating a relatively stable cohort.

In this context, CHWs work as generalists, meaning that they provide a broad range of services in the community. The management of NCDs forms only a part of the package of services offered by CHWs among others. The NCD-related roles they performed ranged from the distribution of medication to measuring of blood pressure and advice about diets. These roles are a clear indication of CHWs’ efforts in assisting clients to better self-manage their conditions. Self-management is a critical component of chronic disease management (Wagner et al. 2001). However, offering a broad range of services can inhibit their ability to be efficient. Thus, there is a need to develop a set of realistic roles that not only cater to the need of the community but also consider the realities of CHWs.

Training, together with technical and material support, is regarded as one of the crucial factors in CHW performance (Dal Poz et al. 2007; Lehmann & Sanders 2007). Despite their designated roles in NCDs, only 52% of the CHWs reported to have received formal training in this domain. In addition, there was a lack of standardisation of both trainers and training duration. Inconsistencies in the training result in discrepancies in practice, which may contribute to differences in care between and within organisations. Models of standardised and structured approaches to CHW training in NCDs have been documented elsewhere (Love et al. 2004), and could serve as a basis for developing programmes in South Africa.

To retain knowledge, follow-up or refresher training is necessary. In the study, a few CHWs received refresher training. In the absence of refresher training, knowledge and the ability to perform specific tasks can be quickly lost (Singh & Sachs 2013). In a study in Nepal, 3 days of additional training of CHWs once a year was found to improve the quality of services provided (Curtale et al. 1995).

The work of CHWs requires them to be knowledgeable about the most prevalent diseases in the community. Overall, knowledge scores were poor for both diabetes and hypertension. This lack of knowledge has implications for their roles such as the provision of information, advising on the dietary intake and facilitation of support groups. Cherrington et al. in their study found that CHWs’ lack of knowledge regarding diabetes provided misguided information to patients (Cherrington et al. 2008). Misinformation has the potential to lead to serious medical consequences. However, Cherrington et al. warn that the scope of information that CHWs are responsible for should be re-examined to avoid overburdening CHWs.

The multivariate analysis showed that having an NCD and regular supervision was associated with better knowledge scores. Such findings suggest that CHWs with a condition could also act as expert patients, and because they have the condition, they may be more motivated to acquire knowledge. Supervision is well established as a positive influence on CHW performance and has been associated with improved diabetes and hypertension knowledge when coupled with training (Labhardt et al. 2010).

The results of this study suggest that there are multiple potential sources of NCD-related knowledge apart from formalised training; acknowledging these processes of knowledge acquisition is therefore essential in the debate on the capacity building of CHWs. These alternative sources of knowledge open the possibility for informal training where knowledge transfer occurs through supervisor-led induction and peer-led education. Although relatively few respondents (*n* = 18; 25%) indicated that they had received induction from other CHWs, in a previous study which observed daily activities of CHWs, it became apparent that peer-to-peer training or peer-led education is an essential source of learning and knowledge transfer (Tsolekile et al. 2014). This approach offers CHWs an opportunity to learn from each other outside hierarchical settings, which often is the case in formal training settings. Although peer training requires facilitation skills and the identification of suitable performers, it still shows some promise. Furthermore, peer-led approaches to training may be another way of supporting and supplementing standardised training (Josiah Willock et al. 2015).

Many have viewed effective and regular supervision as a strategy to assist with work-related challenges experienced by CHWs (Brownstein et al. 2005; Dal Poz et al. 2007). Others have reported that the quality of support and supervision provided to CHWs, together with the promotion of their safety and well-being, resulted in improved motivation and performance (Jaskiewicz & Tulenko 2012). Also, supervision can assist in identifying and correcting poor practices, thus aiding in building the capacity of CHWs. In this study, CHWs reported that they were under regular supervision and supervisors, who are professional nurses, often observed their work. It has been shown that diversity in supervision approaches and the quality of supervision yielded a more significant impact compared to the frequency of supervision (Brownstein et al. 2005).

### Limitations of the study

Although this study provided noteworthy findings, the results should be interpreted with caution, especially about the generalisability of the findings. It was conducted in only two NGOs that deal with NCDs such as diabetes and hypertension as a part of a comprehensive package of care for a range of diseases, leaving out NGOs that may predominantly work with diseases such as HIV. The Western Cape, where the study was undertaken, is well resourced compared to other provinces in South Africa; this could be reflected in the quality of the supervision and the ability to access training opportunities from other institutions. Finally, the sample size was relatively small, limiting the possibility of disaggregated analyses. Nevertheless, the study provides insight into community-based NCD care, as well as training, supervision, knowledge and roles of CHWs who are responsible for providing NCD services in communities.

## Recommendations

The findings have specific implications for the community prevention, management and control of NCD at the community level. Firstly, it is recommended that the scope of practice of CHWs should be defined, and their roles should consider the context and characteristics of CHWs. Secondly, standardised NCD training should be provided to all CHWs and NGOs, and this training should ideally be included as part of core modules in basic generalist training. Thirdly, training should be linked to structured in-service refresher programmes to ensure that knowledge from initial training is retained. Furthermore, this training needs to be of sufficient duration and should adopt appropriate methods. To enhance the roles of CHWs and to strengthen CHW programmes, work-based learning and support from supervisors as well as peers should be considered. Lastly, the study suggests the need to consider and further explore informal processes of knowledge acquisition such as supervisor and peer education, especially in contexts where formal training programmes are scarce.

## Conclusion

This study suggests a need for an integrated approach to building the capacity of CHWs for NCD care that combines considerations of selection (as an expert patient) with structured training and supportive supervision. This work also provides insight into the need for the standardisation of training material as well as follow-up training that is structured and linked to basic training. Furthermore, the study highlights informal training systems that exist within NGOs, and these need to be considered when designing training systems.
